# Specific gene expression signatures induced by the multiple oncogenic alterations that occur within the PTEN/PI3K/AKT pathway in lung cancer

**DOI:** 10.1371/journal.pone.0178865

**Published:** 2017-06-29

**Authors:** Carmela De Marco, Carmelo Laudanna, Nicola Rinaldo, Duarte Mendes Oliveira, Maria Ravo, Alessandro Weisz, Michele Ceccarelli, Elvira Caira, Antonia Rizzuto, Pietro Zoppoli, Donatella Malanga, Giuseppe Viglietto

**Affiliations:** 1 Dipartimento di Medicina Sperimentale e Clinica, Università “Magna Graecia”, Catanzaro, Italia; 2 Biogem scarl, Instituto di Rihe Genetiche “Gaetano Salvatore”, Ariano Irpino, Italia; 3 Laboratorio di Medicina Molecolare e Genomica, Facoltà di Medicina e Chirurgia, Università di Salerno, Baronissi, Italia; 4 Dipartimento di Studi Biologici e Ambientali, Università del Sannio, Benevento, Italia; 5 Dipartimento di Scienze Mediche e Chirurgiche, Università “Magna Graecia”, Catanzaro, Italia; Centro Nacional de Investigaciones Oncologicas, SPAIN

## Abstract

Hyperactivation of the phosphatydil-inositol-3' phosphate kinase (PI3K)/AKT pathway is observed in most NSCLCs, promoting proliferation, migration, invasion and resistance to therapy. AKT can be activated through several mechanisms that include loss of the negative regulator PTEN, activating mutations of the catalytic subunit of PI3K (PIK3CA) and/or mutations of AKT1 itself. However, number and identity of downstream targets of activated PI3K/AKT pathway are poorly defined. To identify the genes that are targets of constitutive PI3K/AKT signalling in lung cancer cells, we performed a comparative transcriptomic analysis of human lung epithelial cells (BEAS-2B) expressing active mutant AKT1 (AKT1-E17K), active mutant PIK3CA (PIK3CA-E545K) or that are silenced for PTEN. We found that, altogether, aberrant PI3K/AKT signalling in lung epithelial cells regulated the expression of 1,960/20,436 genes (9%), though only 30 differentially expressed genes (DEGs) (15 up-regulated, 12 down-regulated and 3 discordant) out of 20,436 that were common among BEAS-AKT1-E17K, BEAS-PIK3CA-E545K and BEAS-shPTEN cells (0.1%). Conversely, DEGs specific for mutant AKT1 were 133 (85 up-regulated; 48 down-regulated), DEGs specific for mutant PIK3CA were 502 (280 up-regulated; 222 down-regulated) and DEGs specific for PTEN loss were 1549 (799 up-regulated, 750 down-regulated). The results obtained from array analysis were confirmed by quantitative RT-PCR on selected up- and down-regulated genes (n = 10). Treatment of BEAS-C cells and the corresponding derivatives with pharmacological inhibitors of AKT (MK2206) or PI3K (LY294002) further validated the significance of our findings. Moreover, mRNA expression of selected DEGs (SGK1, IGFBP3, PEG10, GDF15, PTGES, S100P, respectively) correlated with the activation status of the PI3K/AKT pathway assessed by S473 phosphorylation in NSCLC cell lines (n = 6). Finally, we made use of Ingenuity Pathway Analysis (IPA) to investigate the relevant BioFunctions enriched by the costitutive activation of AKT1-, PI3K- or PTEN-dependent signalling in lung epithelial cells. Expectedly, the analysis of the DEGs common to all three alterations highlighted a group of BioFunctions that included Cell Proliferation of tumor cell lines (14 DEGs), Invasion of cells (10 DEGs) and Migration of tumour cell lines (10 DEGs), with a common core of 5 genes (ATF3, CDKN1A, GDF15, HBEGF and LCN2) that likely represent downstream effectors of the pro-oncogenic activities of PI3K/AKT signalling. Conversely, IPA analysis of exclusive DEGs led to the identification of different downstream effectors that are modulated by mutant AKT1 (TGFBR2, CTSZ, EMP1), mutant PIK3CA (CCND2, CDK2, IGFBP2, TRIB1) and PTEN loss (ASNS, FHL2).

These findings not only shed light on the molecular mechanisms that are activated by aberrant signalling through the PI3K/AKT pathway in lung epithelial cells, but also contribute to the identification of previously unrecognised molecules whose regulation takes part in the development of lung cancer.

## Introduction

Lung cancer is the most frequent cause of cancer-related deaths worldwide [1, 2]. Lung cancer comprises two main groups that include small-cell lung cancer (SCLC) and non-small-cell lung cancer (NSCLC)[1], of which the latter accounts for 80–85% of cases.

At present, five-year survival of lung cancer patients is low [3], because it is often detected in advanced stages [4]. For this reason a more complete understanding of the molecular origins of the disease may help contribute to improve therapeutic regimens.

The phosphatidylinositol 3-kinase (PI3K) signaling cascade plays a critical role in the initiation and/or progression of NSCLC [5–11]. This pathway regulates multiple cellular processes that are relevant in the growth and progression of lung cancer cells including cell proliferation, migration, apoptosis and angiogenesis [12]. The protein kinase B (PKB), also known as AKT, is an important mediator of the PI3K pathway, representing the end-point of signaling elicited by several growth factors and cytokines [13].

AKT is activated by recruitment to cell membrane through the binding of its PH domain to 3′-phosphorylated phosphatidyl-inositols generated by PI3K and subsequent phosphorylation at T308 and S473 [14–16]. Conversely, dephosphorylation of the 3′ position of phosphatidyl-inositols exerted by the lipid phosphatase PTEN attenuates AKT activation [17].

Upon its activation, AKT phosphorylates a number of proteins that include kinases (GSK-3α/β, IκKalpha), cell cycle inhibitors (CDKN1A, CDKN2B), transcription factors (FOXO1, FOXO3a) and/or proteins involved in apoptosis (Bad, procaspase-9) [18]. In turn, AKT-dependent phosphorylation regulates stability and/or cellular localization of its substrates [19–22]. Interestingly, numerous direct and indirect substrates of AKT and/or of the PI3K pathway are transcription factors [23]. PI3K- and/or AKT-dependent phosphorylation of transcription factors frequently results in the modulation of their activity [18, 24, 25].

In this manner, genes that are regulated by the PI3K/AKT pathway may contribute to mediate the multiple activities that are under the control of this pathway.

AKT is frequently activated in NSCLC [26–28], with active AKT being associated with higher grade, more advanced stage [8] and lymph node metastasis [29]. Accordingly, NSCLC patients with tumors showing high AKT activity present decreased survival [29, 30].

In NSCLC, AKT activation may result from distinct and often mutually exclusive events that include activating mutations or increased expression of one or more of AKT isoforms (AKT1, AKT2 or AKT3) or of their upstream regulators such as KRAS or PIK3CA or loss of negative regulators (i.e. PTEN) [8, 31]. However, whereas the effects of AKT activation on the levels and/or the localization of specific substrates have been largely studied, so far a global analysis of the genes regulated by the different activated members of the PI3K/AKT signaling is missing.

In this manuscript we identified the panel of genes that are regulated by aberrant PI3K/AKT signaling in lung epithelial cells, by analysis of gene profiles that are induced by specific alterations that include PIK3CA mutations, AKT1 mutations and PTEN loss. The findings reported here contribute to shed light on the molecular mechanisms that are activated by aberrant signalling through the PI3K/AKT pathway in lung epithelial cells, as well as to identify molecules, previously unrecognised, whose regulation contributes to the tumorigenic activity of this pathway in lung epithelial cells.

## Materials and methods

### Cell culture

BEAS-2B, immortalized human bronchial epithelial cell line was purchased from Chambrex (Milan, Italy) and grown according to the manufacturer’s protocol.

Human lung cancer cell lines (NCI-H23, NCI-H292, NCI-H522, NCI-H460, A549, NCI-H226) were purchased from ATCC-LGC Promochem (South West London, UK) and maintained in RPMI 1640 (Lonza Walkersville Inc., MD) supplemented with 10% fetal bovine serum (Sigma-Aldrich, St.Luis, MO, USA) and 100 units/ml penicillin-streptomycin (Lonza Walkersville Inc.).

### RNA extraction

Total RNA isolation from cells and clinical samples was performed with Trizol Reagent (Invitrogen, Carlsbad, CA, USA) according to the manufacturer’s protocol. RNA integrity was checked by use of denaturing agarose gel electrophoresis.

### RNA profiling analysis

RNA concentration was determined with Nanodrop spectrophotometer (Nano-Drop, Wilmington, Germany) and its quality was assessed with Agilent 2100 Bioanalyzer (Agilent Technologies, Santa Clara, CA). For each sample, 500 ng of total RNA were used to synthesize biotinylated cRNA with Illumina RNA Amplification Kit (Ambion, Austin, TX). Synthesis was carried out according to the manufacturers’ instructions. cRNA concentration and the quality were assessed out as described above. From each sample, three technical replicates were produced and 750 ng cRNA were hybridized for 18h to Human HT-12_V3_0_R1 Expression BeadChips (Illumina, San Diego, CA) as described earlier [32]. Hybridized chips were washed and stained with streptavidin-conjugated Cy3 (GE Healthcare, Milan, Italy). BeadChips were dried and scanned with an Illumina Bead Array Reader (Illumina).

### Data analysis

Raw data from Array hybridization were loaded into and analyzed by Gene-Spring GX 14.5 (Agilent Technologies). DEGs were obtained performing ono-to-one comparison of the RNA profiles of BEAS-C derivatives (BEAS-AKT1-E17K, BEAS-PIK3CA-E545K and BEAS-shPTEN) compared to control BEAS-C cells, experiments were performed in triplicates. DEGs were selected on the basis of absolute fold-change (≥ 2) and statistical significance (p<0.01). Statistical analysis was performed by moderated t-test. P-values were corrected by Benjamini-Hochberg (B–H) FDR correction (q-value cut-off 0.01) [33]. Ingenuity Pathway Analysis (IPA, Ingenuity Systems) was used to evaluate the functional behaviour of DEGs in terms of Biological Processes and Molecular Function, Development Function, Disease and Disorder. The degrre of enrichment of the IPA analysis was performed by the Fisher’s exact test (p ≤ 0.05).

### Real time RT-PCR (q-PCR)

Total RNA was prepared as described in [34, 35]. Q-RT-PCR was performed using the Power SYBR Green PCR Master Mix in an ABI Prism 7300 thermocycler (Applied Biosystems, Foster City, CA, USA). cDNAs were synthesized from 1 μg of total RNA using QuantiTect Reverse Trascription (Qiagen, Venlo, The Netherlands). Normalization was performed to GAPDH mRNA content. The relative amounts of mRNA or DNA were calculated by the comparative cycle threshold (CT) method by Livak and Schmittgen [36]. Primers for Q-RT-PCR are reported in S1 Table.

### Western blot and antibodies

Whole cell protein extracts were prepared by homogenizing cells in NP-40 lysis buffer (10 mM Tris–HCl (pH 7.5), 150 mM NaCl, 1% NP-40) containing protease and phosphatase inhibitors. Western blot analysis was carried out by standard methods; chemioluminescence was detected with Alliance Mini WL2M system (Uvitec, Cambridge, UK). Anti-phospho-AKT (Ser473) (Rabbit, #4058, 1:1000) and anti-AKT1 (Rabbit, #2938, 1:1000) were purchased from Cell Signaling Technology (Denver, MA, USA); anti-actin (goat, sc-1616, 1:3000) was purchased from Santa Cruz Biotechnology (Santa Cruz, CA).

### Statistical analysis

RT-PCR data are expressed as means±SD of at least three independent experiments conducted in triplicates as indicated in the text. Statistical significance was evaluated by One-way, Two-way ANOVA or *t-*test tests as indicated in the figure legends. Statistical significance was indicated as follows: p≤ 0.05 (*), p≤ 0.01 (**), p≤ 0.001 (***) and p≤ 0.0001 (****).

## Results and discussion

### Identification of PI3K/AKT-dependent gene expression signature in lung epithelial cells

The purpose of this study was to identify genes that are modulated by aberrant PI3K/AKT signalling in lung epithelial cells, and for this reason, may represent novel determinants of the pro-tumorigenic activity of the PI3K/AKT pathway. To this aim, we have genetically modified bronchial epithelial cells that recapitulate the most frequent alterations in genes within the PI3K/AKT pathway. As model cell line we chose BEAS-2B cells, an immortalised bronchial epithelial cell line of human origin that do not support tumour growth [37].

BEAS-2B cells were engineered with an ampty vector or to express gain-of-function mutations of PIK3CA (PIK3CA-E545K), of AKT1 (AKT1-E17K) or were silenced for PTEN. These cells have been denoted BEAS-C, BEAS-AKT1-E17K, BEAS-PIK3CA-E545K and BEAS-shPTEN, and have already been characterised for their proliferative, migratory/invasive and tumorigenic properties [38, 39]. Hereafter BEAS-AKT1-E17K, BEAS-PIK3CA-E545K and BEAS-shPTEN will be referred to BEAS-2B derivatives.

The presence of exogenous mutant PIK3CA and AKT1 proteins or of the endogenous wild-type PTEN protein in transduced cells as well as the activation of PI3K/AKT signalling were already determined [38, 39].

To identify genes that are targets of constitutive signalling of PI3K/AKT in lung cancer cells, we have analysed gene expression profiles of BEAS-C cells and derivatives. Expression values of the mRNAs obtained were filtered for log2 fold change ≥2. The transcriptome of BEAS-PIK3CA-E545K cells has already been analysed in a previous study, using a slightly lower log2 fold change threshold of 1.5 [8] and reanalysed with the more selective threshold used in this manuscript to be compared with BEAS-AKT1-E17K and BEAS-shPTEN cells.

Analysis of results allowed the definition of 3 lists of differentially expressed gene probes (See S1–S3 Files).

**Fig 1:**
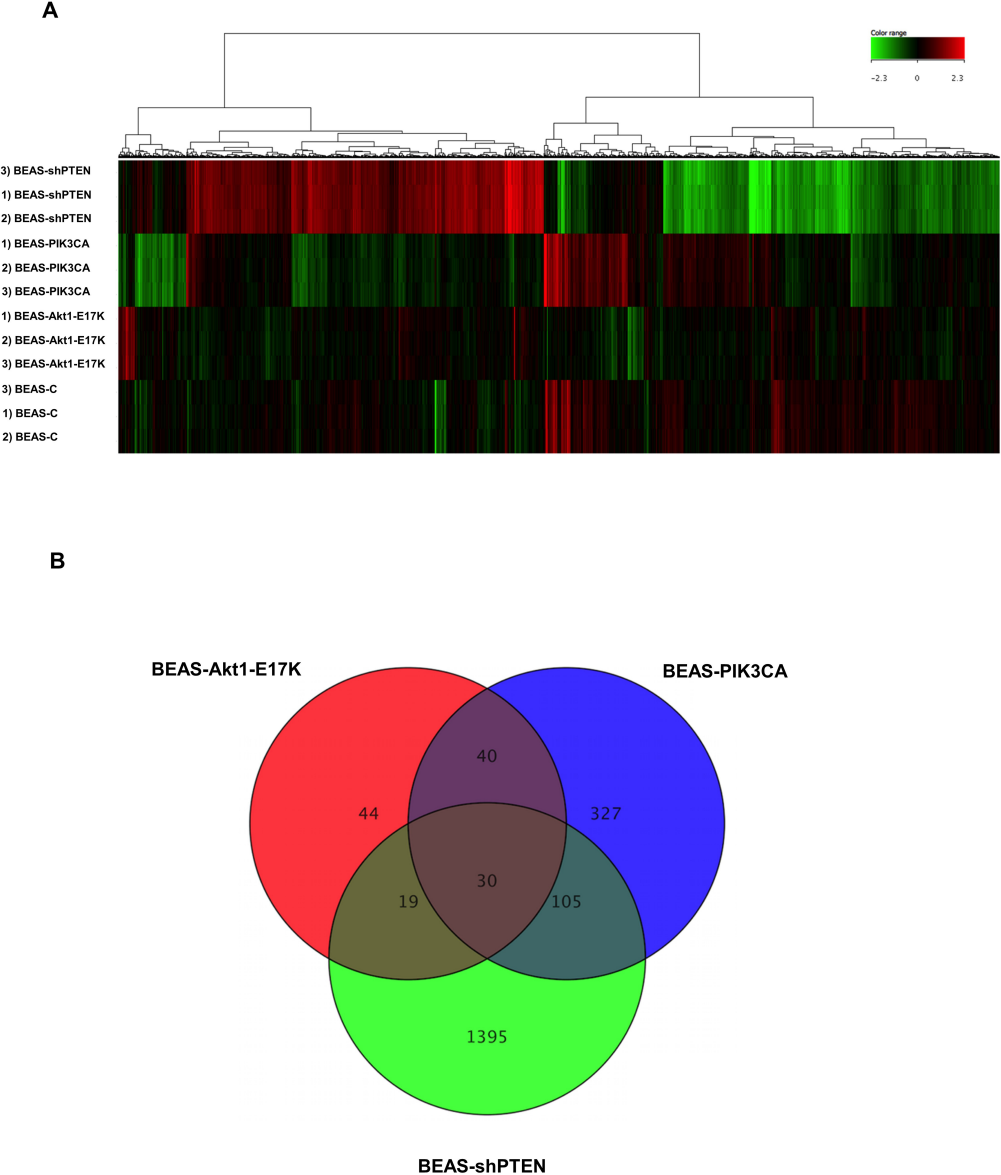
Array analysis of genes regulated by the PI3K/AKT pathway in lung epithelial cells. (A) Heatmap representation of expression values of DEGs comparing BEAS-AKT1-E17K, BEAS-PIK3CA-E545K and BEAS-shPTEN to BEAS-C control cells. Hierarchical clustering analysis was perfomed with Euclidean metric distance and Ward’s linkage rule. (B) The Venn diagram shows the number of common, specific and exclusive DEGs.

As shown, we identified 133 DEGs in cells expressing mutant AKT1 (85 of which were up-regulated and 48 were down-regulated), 502 DEGs in cells expressing mutant PIK3CA (280 of which were up-regulated and 222 were down-regulated) and 1549 DEGs in cells silenced for PTEN (799 of which were up-regulated and 750 were down-regulated). See the heatmap in Fig 1A for graphic representation of the observed differences in gene expression. As shown the gene expression profile resulting by PTEN silencing was remarkably different from those induced by mutant AKT1 and PIK3CA, respectively. Microarray raw data for all probes have been deposited in the ArrayExpress database (www.ebi.ac.uk/arrayexpress) under accession number E-MTAB-5286.

Once we identified the genes that are regulated by aberrant PI3K/AKT signaling in human lung cells, we proceeded to compare DEG lists in order to identify genes whose expression was modified in cells expressing mutant AKT1, mutant PIK3CA, PTEN loss and/or a combination thereof. Being regulated by alterations in all three conditions, these genes are expected to be the main mediators of aberrant PI3K/AKT signalling in transformed lung epithelial cells, most of which have not apparently been recognised yet.

As shown in the Venn diagram of Fig 1B, DEGs that are regulated by active AKT1, active PIK3CA and PTEN loss (hereafter common DEGs) were 30, though only 27 showed similar behaviour in all three conditions (15 up-regulated, 12 down-regulated and 3 discordant). The complete list of common DEGs with fold-change values is shown in Table 1. Beside the group of 27 common DEGs, we also found, by two-by-two comparison, that 106 DEGs (48 up-regulated, 40 down-regulated and 18 discordant) were common between BEAS-PIK3CA-E545K and BEAS-shPTEN, 41 DEGs (25 up-regulated, 16 down-regulated) were common between BEAS-AKT1-E17K and BEAS-PIK3CA-E545K cells, and 19 DEGs (13 up-regulated, 5 down-regulated and 1 discordant) were common between BEAS-AKT1-E17K and BEAS-shPTEN cells (Fig 1B). See S4–S6 Files.

**Table 1:** Common DEGs showing the highest values of fold change.

TABLE 1	Illumina_Gene	Illumina Probe_Id	FC^a^ BEAS-AKT1-E17K	FC^a^ BEAS-PIK3CA-E545K	FC^a^ BEAS-shPTEN	MEAN FC^a^
**UP-REGULATED GENES**	S100P	ILMN_1801216	5.5	4.4	18.2	9.4
	GDF15	ILMN_2188862	29.1	5.4	24.2	19.6
	PTGES	ILMN_1713829	7.0	8.9	2.1	6.0
	KRT81	ILMN_1801442	7.5	6.1	3.6	5.7
	LCN2	ILMN_1692223	3.1	7.9	4.4	5.1
	DUSP5	ILMN_1656501	3.4	7.6	2.8	4.6
	ATF3	ILMN_2374865	2.1	5.6	4.8	4.2
	GADD45A	ILMN_2052208	2.8	4.9	3.3	3.7
	UPP1	ILMN_1798256	3.3	2.3	3.6	3.1
	HBEGF	ILMN_2121408	2.3	4.1	2.9	3.1
	PHLDA1	ILMN_1687978	3.0	3.6	2.1	2.9
	SDC4	ILMN_1663042	2.8	3.2	2.6	2.9
	CDKN1A	ILMN_1784602	3.2	2.0	2.3	2.5
	CYSRT1	ILMN_1814106	2.6	2.6	2.4	2.5
	KRT17P3	ILMN_1653934	2.3	2.1	2.6	2.3
**DOWN-REGULATED GENES**	HS.570308	ILMN_1900110	-2.6	-2.8	-8.3	-4.6
	PEG10	ILMN_2297626	-2.3	-4.8	-5.9	-4.3
	IGFBP3	ILMN_1746085	-3.6	-5.2	-2.9	-3.9
	GJA1	ILMN_1727087	-2.5	-4.3	-4.5	-3.8
	SGK	ILMN_1702487	-3.7	-3.6	-3.7	-3.7
	BMF	ILMN_2308338	-4.2	-3.5	-2.4	-3.4
	BEX1	ILMN_2234697	-2.3	-3.8	-2.5	-2.9
	MARCKS	ILMN_1807042	-2.6	-2.4	-3.4	-2.8
	VWA5A	ILMN_1682996	-2.8	-3.2	-2.2	-2.7
	GFOD1	ILMN_1778240	-2.3	-2.4	-2.7	-2.5
	HS.105791	ILMN_1840316	-2.0	-2.1	-3.0	-2.3
	GFRA1	ILMN_1898518	-2.3	-2.4	-2.3	-2.3
**DISCORDANT**	EXOG	ILMN_1709747	2.09	3.4	-2.69	N.A.
	PLAC9	ILMN_1790859	-2.46	-3.19	2.34	N.A.
	LOC441019	ILMN_1761281	2.66	5.5	-3.12	N.A.

^**a**^ FC = Fold Change N. A.: not applicable

Finally, we identified DEGs that were exclusive to single gene alterations (hereafter exclusive DEGs). Exclusive DEGs were 44 in BEAS-AKT1-E17K (31 up-regulated, 13 down-regulated), 327 in BEAS-PIK3CA-E545K (181 up-regulated, 146 down-regulated) and 1395 in BEAS-shPTEN (713 up-regulated, 682 down-regulated). The complete lists of exclusive DEGs are reported in See S7–S9 Files. The exclusive DEGs with the highest values of log2 fold change are listed in Tables 2–4.

**Table 2:** Exclusive DEGs showing the highest values of fold change specific for mutant AKT1.

TABLE 2	GENE	log2 FC	q value
**UP-REGULATED GENES**	CKB	4.7	3.86E-13
	IGFL1	3.4	5.93E-12
	CLEC2D	3.4	1.96E-11
	CPA4	3.3	1.96E-11
	GPRC5C	3.1	5.37E-10
	KRT16	2.9	9.03E-11
	SERPINE2	2.8	1.40E-10
	SERPINA3	2.7	3.69E-09
	LCN15	2.6	7.61E-10
	ATP5G1	2.6	1.37E-09
**DOWN-REGULATED GENES**	STAT2	-2.7	4.56E-10
	COPG2	-2.5	4.93E-08
	TAF15	-2.5	2.97E-09
	GRAMD2	-2.4	4.46E-09
	TAF15	-2.2	3.02E-08
	NRCAM	-2.2	1.17E-07
	FSTL1	-2.2	3.80E-08
	PDE5A	-2.1	3.78E-08
	WDR19	-2.1	3.10E-07
	OLR1	-2	8.81E-08

**Table 3:** Exclusive DEGs showing the highest values of fold change specific for mutant PIK3CA.

TABLE 3	GENE	log2 FC	q value
**UP-REGULATED GENES**	FOS	14	9.55E-18
	FOSB	12.7	7.09E-17
	HES1	8.3	6.55E-15
	IL1B	7.5	9.54E-16
	LAMB3	6.8	2.57E-15
	BHLHB2	6.6	2.57E-15
	CCND2	6.1	1.77E-14
	IL1A	5.5	1.63E-14
	C1orf116	5.4	1.63E-14
	MT1G	5	6.15E-14
**DOWN-REGULATED GENES**	GPR162	-5.5	1.09E-14
	EFHD1	-4.5	1.10E-13
	PALM	-3.6	8.79E-13
	SNTB1	-3.3	4.19E-12
	BGN	-3.2	7.16E-12
	FBLN1	-3.1	6.71E-12
	ROR2	-3.1	2.62E-11
	CDKN2C	-3.1	5.41E-11
	PMP22	-3.1	5.99E-11
	PRKD1	-3.1	1.20E-10

**Table 4:** Exclusive DEGs showing the highest values of fold change specific for PTEN loss.

TABLE 4	GENE	log2 FC	q value
**UP-REGULATED GENES**	ASNS	8.6	6.94E-16
	PCK2	8	3.54E-16
	IFI27	7.1	7.17E-15
	PSAT1	5.9	2.63E-15
	LARP6	5.5	1.86E-14
	SDSL	5.4	1.95E-14
	STC2	5.3	8.84E-15
	BEX2	5.3	9.83E-15
	ASS1	5.2	9.27E-14
	C6orf48	5.2	3.17E-13
**DOWN-REGULATED GENES**	INSIG1	-10.6	4.37E-17
	TFRC	-10.2	4.37E-17
	USP9X	-6.9	7.34E-16
	SC4MOL	-6.7	1.01E-15
	CYP1B1	-6.2	3.10E-15
	SC4MOL	-5.9	6.77E-15
	LOC728499	-5.7	3.04E-14
	LPP	-5.4	3.68E-14
	ZAK	-5.3	1.95E-14
	LOC730432	-5.3	6.79E-15

These results indicate that, altogether, aberrant PI3K signalling elicited in lung epithelial cells by mutant PIK3CA, mutant AKT1 or PTEN loss regulated the expression of 1,960/20,436 genes (9%), though only 27 DEGs out of 20,436 were common to all three alterations (0.1%).

### Validation of DEGs in BEAS-C cells and derivatives

**Fig 2:**
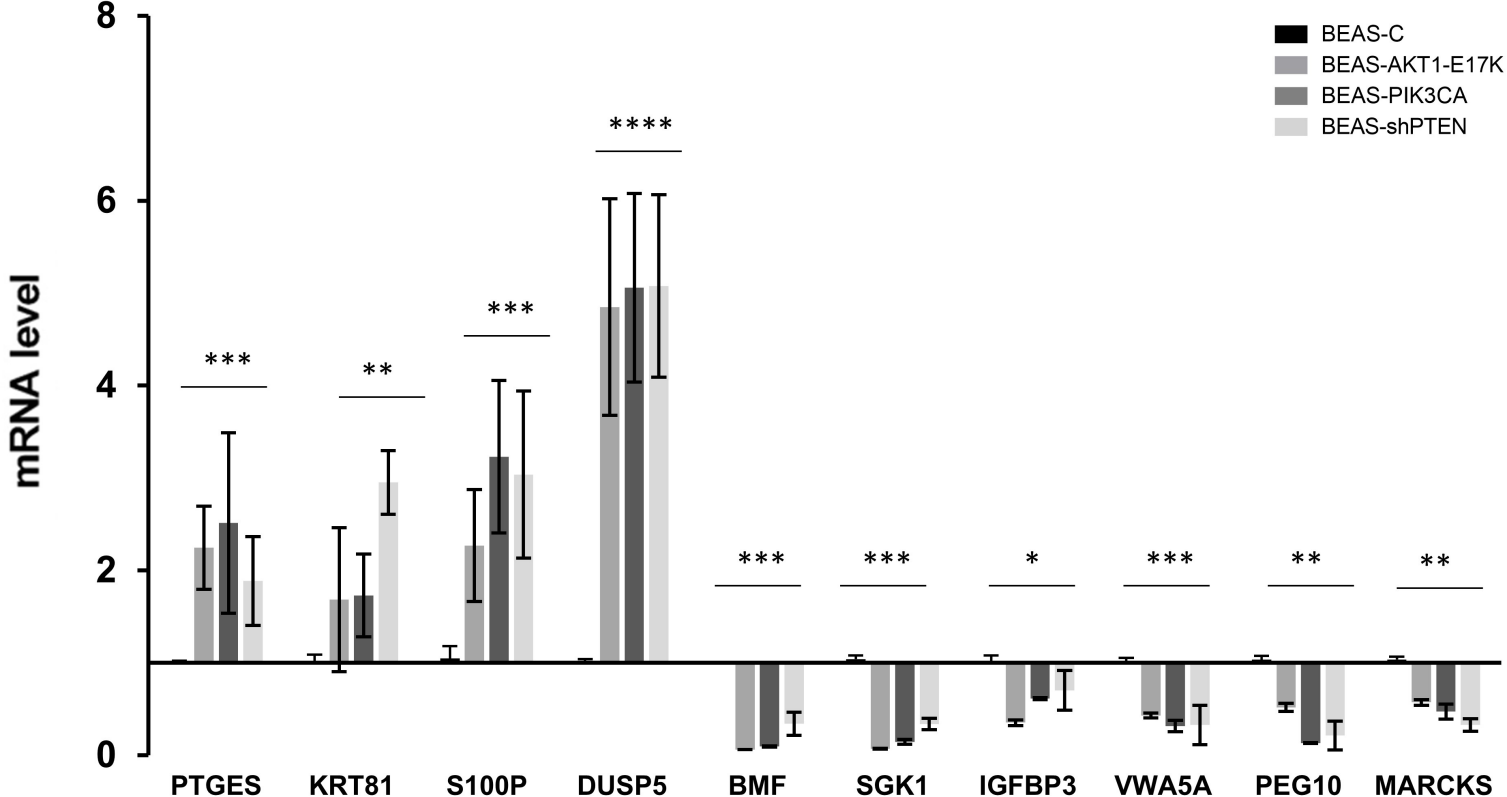
Validation of array results by quantitative RT-PCR analysis. **(A)** Analysis of 10 representative DEGs in BEAS-C and derivatives. Bars are mean ± SD of 4 independent experiments performed in triplicate. Statistical analysis was performed by One-way ANOVA test and Dunnett's test for each gene, p-values as indicated.

We confirmed the results obtained from the array analysis by quantitative RT-PCR on a selected panel of up-regulated (PTGES, KRT81, S100P, DUSP5) and down-regulated genes (BMF, SGK1, IGFBP3, VWA5A, PEG10, MARCKS) (Fig 2). All DEGs validated by qRT-PCR were confimed to be statistically significant, as indicated in the Fig 2.

**Fig 3:**
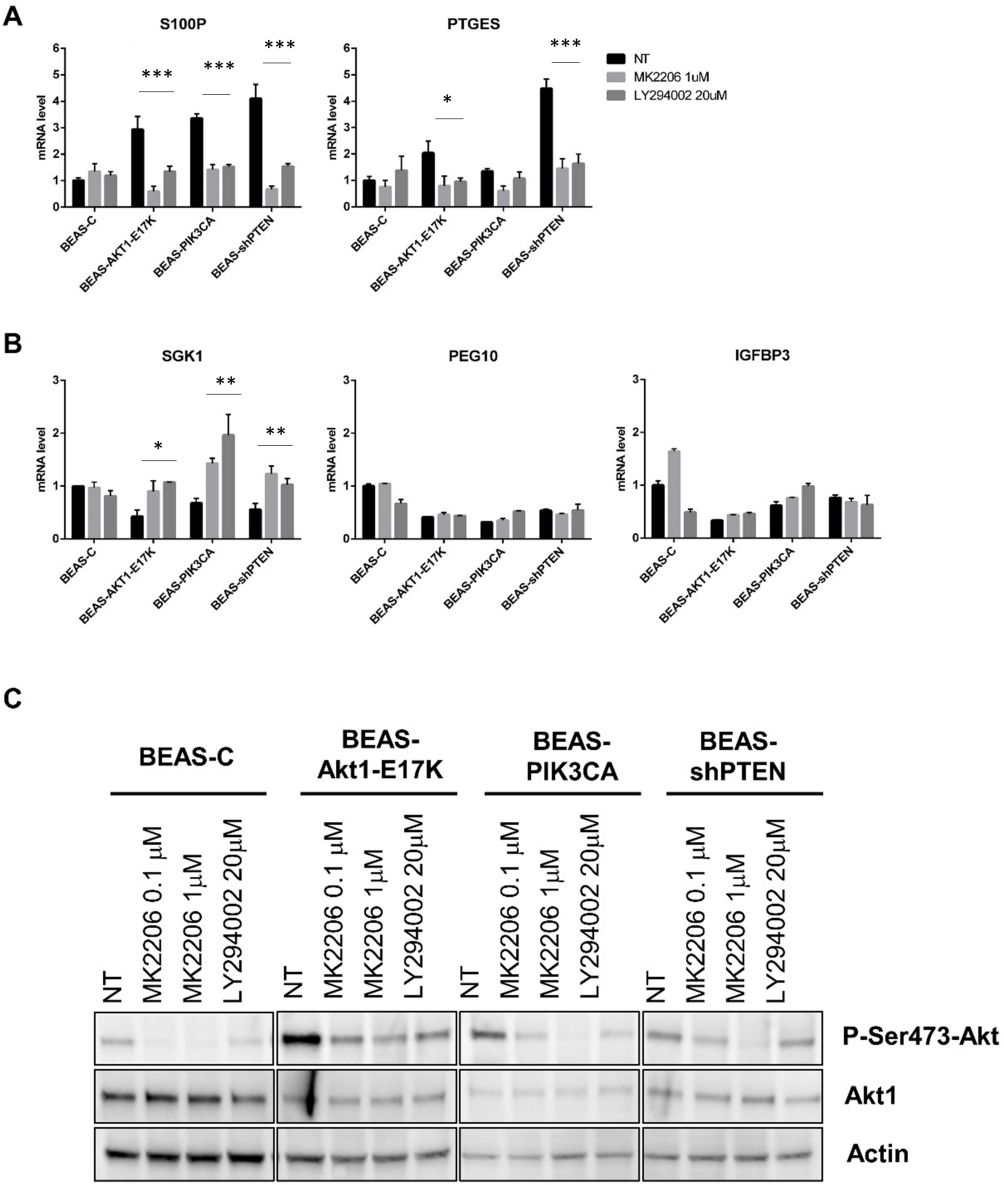
Analysis of DEG expression in BEAS-C and derivatives cells treated with pharmacological inhibitors of the PI3K/AKT pathway. BEAS-C cells and derivatives were treated for 24 hours with the indicated doses of MK2206 and LY294002, respectively. **(A)** The graphs show the mRNA levels (mean ± SD) of the indicated up-regulated genes in BEAS-2B cells and derivatives. **(B)** The graphs show the mRNA levels (mean ± SD) of the indicated down-regulated genes in BEAS-2B cells and derivatives. Bars are mean ± SD of 3 independent experiments conducted in triplicate, statistical analysis that was performed by Two-way ANOVA and Dunnett's test, p-values as indicated **(C)** Immunoblot analysis of Phospho-AKT (Ser 473), AKT1 and actin in BEAS-C cells and derivatives.

Subsequently, we used pharmacological inhibitors of AKT (MK2206) and of PI3K (LY294002) to investigate whether the identified DEGs responded to modulation of the activity of PI3K/AKT pathway. We found that exposure of BEAS-C cells and the corresponding derivatives to MK2206 or LY294002 induced a marked decrease in the mRNA levels of PTGES and S100P (Fig 3A) and a marked increase in the mRNA levels of SGK1 (Fig 3B) in BEAS-AKT1-E17K, BEAS-PIK3CA-E545K and BEAS-shPTEN cells. However, statistical analysis demonstrated that the modulation exerted by pharmacological inhibitors was statistically significant in all three modified cells in the case of S100P and SGK1. Conversely, for PTGES, PEG10 and IGFBP3 we observed an effect that was in line with the predicted modulation of the three DEGs as obtained by array analysis, which was not statistically significant. As control of the experiments we observed that both inhibitors significantly suppressed AKT phosphorylation in BEAS-C and derivative cells (Fig 3C).

### Expression of selected common DEGs in lung cancer cell lines

**Fig 4:**
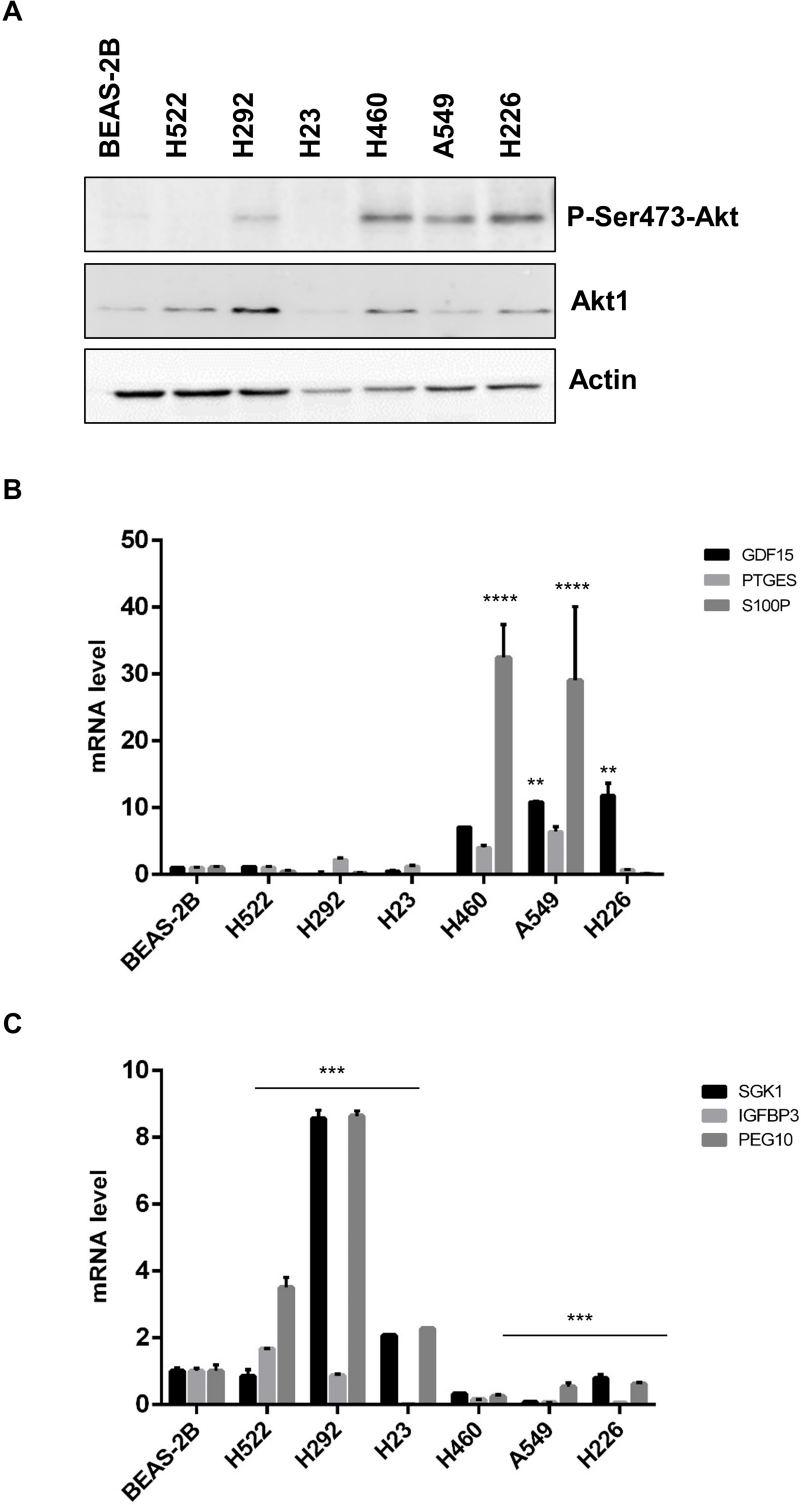
Correlation between the activation status of the PI3K/AKT pathway and DEG expression in NSCLC cell lines. **(A)** Immunoblot analysis of phosphorylated AKT in NSCLC cell lines. AKT1 and actin were used to normalize AKT phosphorylation and protein loading, respectively. **(B)** The graph shows mRNA levels of up-regulated DEGs (GDF15, PTGES, S100P) in NSCLC cell lines. Statistical analysis was performed on 3 independent experiments conducted in triplicate and was performed by One-way ANOVA test for each gene relative to each cell line, p-values as indicated **(C)** The graph shows mRNA levels of three representative down-regulated DEGs (SGK1, IGFBP3, PEG10) in NSCLC cell lines. Bars are mean ± SD of a representative experiment. Statistical analysis was performed on 3 independent experiments conducted in triplicate and was performed by One-way ANOVA test for each cell line, p-values as indicated.

Finally, we investigated the mRNA levels of representative common DEGs that had come out from the array analysis in 6 NSCLC cell lines. The NSCLC cell lines used were NCI-H23, NCI-H292, NCI-H522, NCI-H460, A549 and NCI-H226. To this aim, we analysed the mRNA expression of SGK1, IGFBP3, PEG10, GDF15, PTGES and S100P by quantitative RT-PCR and correlated them with the activation status of the PI3K/AKT pathway as assessed by level of S473 phosphorylation. See Fig 4A.

As shown in Fig 4B, NSCLC cells with low/absent levels of phosphorylated AKT (NCI-H522, NCI-H292, NCI-H23) generally showed lower mRNA levels of GDF15, PTGES and S100P (up-regulated DEGs) than cells with high levels of AKT activity (NCI-H226, NCI-H460, A549) (Fig 4B, p-value as indicated). Similarly, NSCLC cells with low/absent levels of phosphorylated AKT showed significantly high levels of SGK1, IGFBP3, PEG10 (down-regulated DEGs) at difference with what observed in cells with highly phosphorylated AKT (Fig 4C, p-value as indicated).

### Ingenuity Pathway Analysis (IPA) analysis of genes regulated by activation of the PI3K/AKT pathway in lung epithelial cells

In order to search for novel common and/or exclusive effectors of the PI3K/AKT pathway, we used Ingenuity Pathway Analysis (Ingenuity®Systems, IPA, http://www.ingenuity.com) to functionally annotate the lists of DEGs obtained in the experiments described above and to investigate the biological relevance of the transcriptional changes induced by the gain-of-function mutations of AKT1, PIK3CA and/or by the loss of PTEN in lung epithelial cells. To this aim the datasets obtained from common and/or exclusive DEGs were categorized using IPA BioFunctions and we investigated how similar were the biological mechanisms and/or pathways modulated by the alteration of single members (PIK3CA, PTEN, AKT1) within the PI3K/AKT pathway.

**Fig 5:**
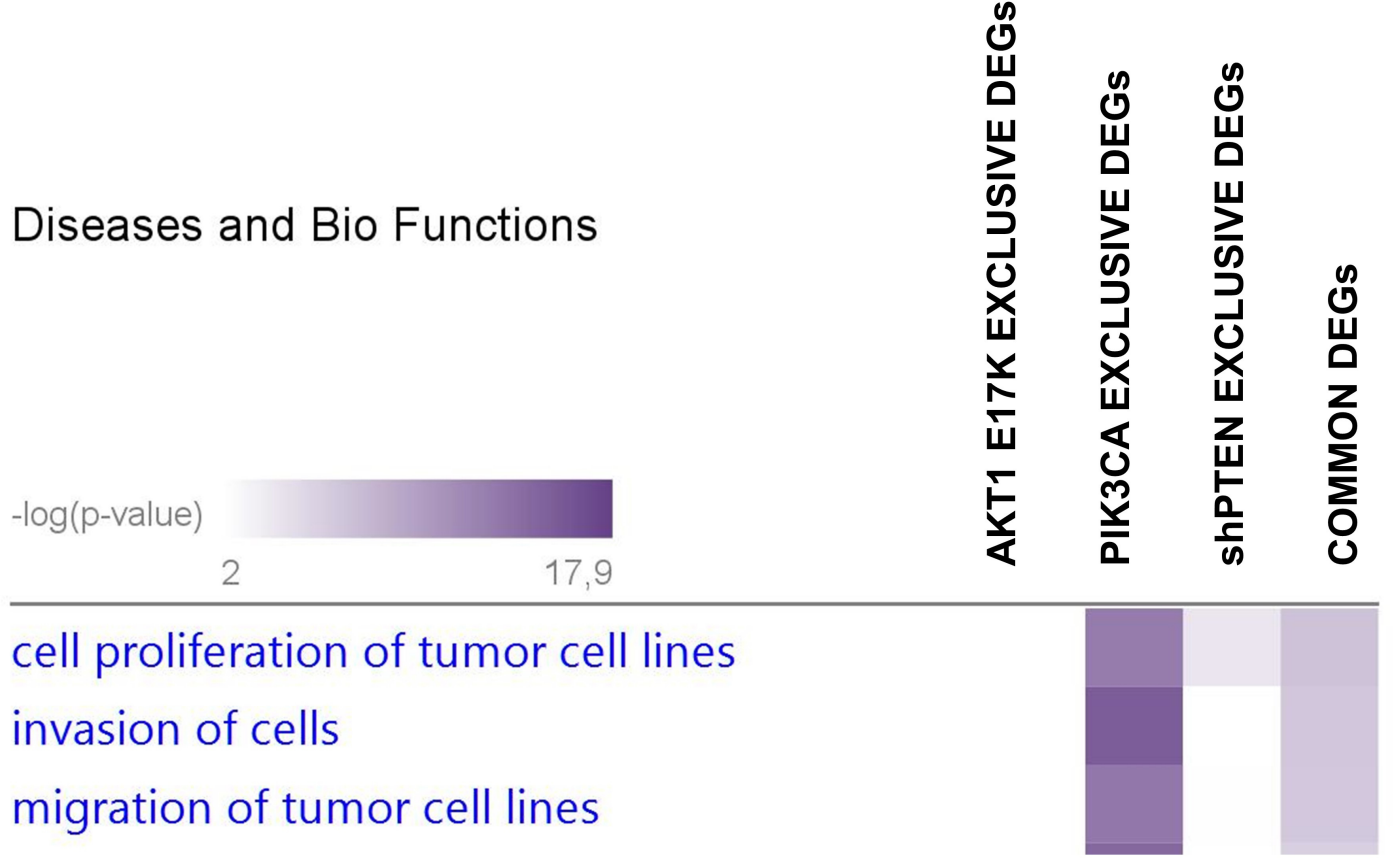
Comparison analysis of Diseases and Bio Functions enriched in BEAS-2B cells expressing constitutively active PIK3CA, AKT1 or interfered for PTEN. IPA heatmap displays the top three Disease and Biofunctions enriched by DEGs that are common among BEAS-AKT1-E17K, BEAS-PIK3CA-E545K and BEAS-shPTEN cells (commons, rightmost column) or that are exclusive for each cell line (AKT1, PIK3CA, shPTEN). Diseases and Bio Functions were sorted for p-value, from the most significant (violet) to the least significant (white) values.

Fig 5 shows a heatmap of the relevant BioFunctions enriched by DEGs common to all three alterations clusterized according to the p-value. Expectedly, relevant BioFunctions that turned out from this analysis included *Cell Proliferation of tumor cell lines* (14 DEGs), *Invasion of cells* (10 DEGs) and *Migration of tumour cell lines* (10 DEGs), all of which are known to be actively regulated by the PI3K/AKT pathway (see Table 5).

**Table 5:** Selected BioFunctions and Diseases enriched by common DEGs.

BioFunctions and Diseases		
Cell proliferation of tumour cell lines	Invasion of cells	Migration of tumour cell lines
ATF3	ATF3	ATF3
CDKN1A	CDKN1A	CDKN1A
DUSP5	GDF15	GDF15
GADD45A	GFRA1	GJA1
GDF15	GJA1	HBEGF
GJA1	HBEGF	IGFBP3
HBEGF	LCN2	LCN2
IGFBP3	MARCKS	PHLDA1
LCN2	S100P	S100P
PEG10	SDC4	SDC4
PHLDA1		
PTGES		
SDC4		
SGK1		

The common DEGs enriched within the indicated BioFunctions are listed in Table 6. We observed that the three BioFunctions contained a common core of 7 genes, namely ATF3 (average log2 fold change of +2.1), CDKN1A (average log2 fold change of +2.5), GDF15 (average log2 fold change of +19.6), GJA1 (average log2 fold change of -3.4), HBEGF (average log2 fold change of +3.1), LCN2 (average log2 fold change of +5.1) and SDC4 (average log2 fold change of +2.9), which are potentially involved in the simultaneous regulation of proliferation, migration and invasion.

**Table 6:** Genes shared by the three BioFunctions and Diseases enriched by common DEGs.

Gene Symbol	Entrez Gene Name	Median FC
ATF3	Activating Transcription Factor 3	4.2
CDKN1A	Cyclin-dependent kinase inhibitor 1A	2.5
GDF15	Growth differentiation factor 15	19.6
HBEGF	Heparin-binding EGF-like growth factor	3.1
LCN2	Lipocalin 2	5.1
SDC4	Syndecan 4	2.9
GJA1	Gap junction protein alpha 1	-3.8

**Fig 6:**
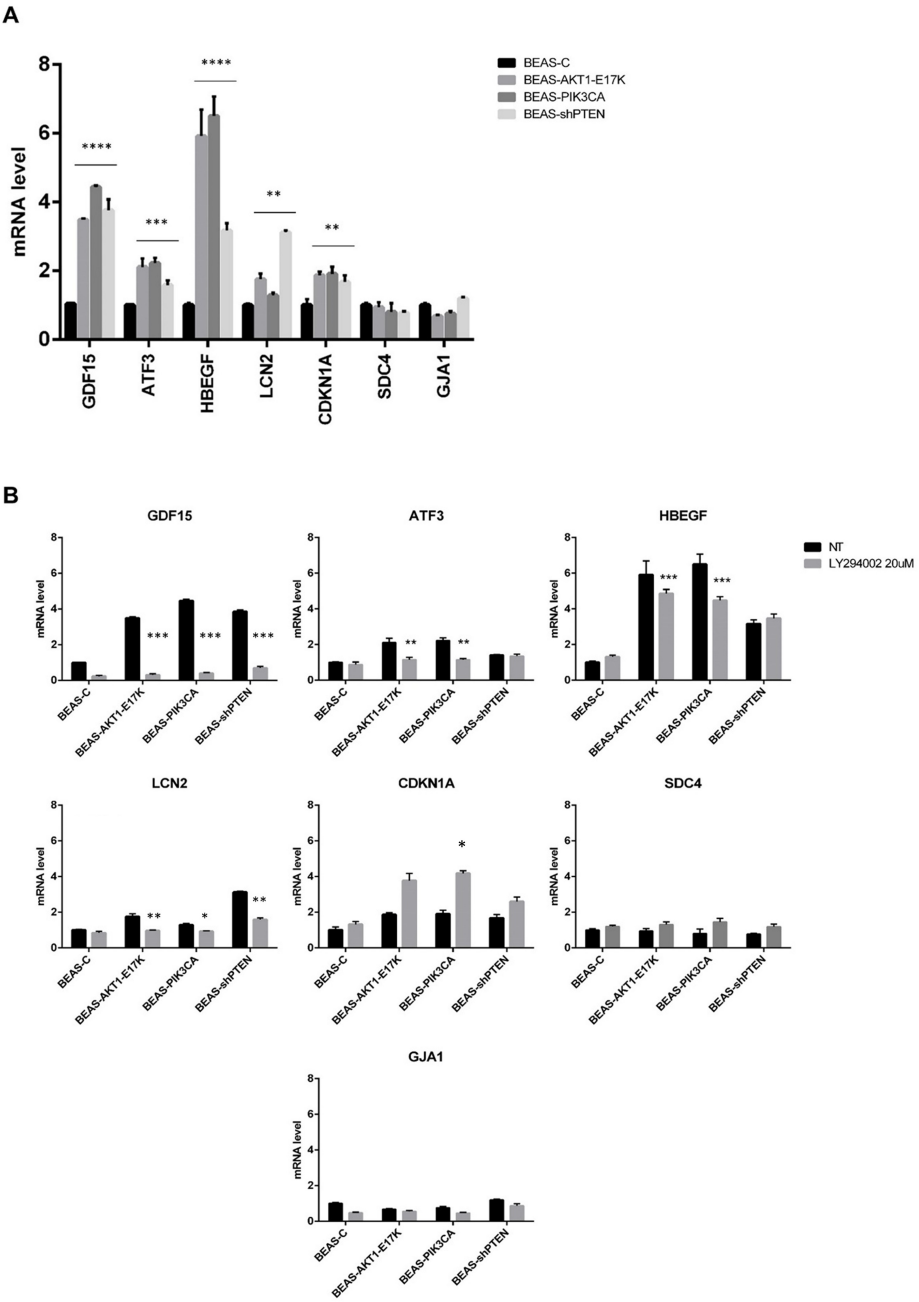
Validation of common DEGs in BEAS-C and derivatives cells treated with pharmacological inhibitors of the PI3K/AKT pathway. **A)** Analysis of 7 common DEGs enriched in BioFunctions in BEAS-C and derivatives. Bars are mean±SD of 3 independent experiments performed in triplicate. Statistical analysis was performed by One-way ANOVA test and Dunnett's test for each gene. p-values were indicated in the figure. **B)** BEAS-C cells and derivatives were treated for 24 hours with the indicated doses of LY294002. Graphs show mRNA levels of the indicated genes in BEAS-C cells and derivatives. Bars are mean±SD of 3 independent experiments conducted in triplicate. Statistical analysis was performed by *t-*test. p-values as indicated in the figure.

To increase the relevance of the common DEGs enriched in the above-mentioned BioFunctions, we validated by qRT-PCR the core of 7 common genes (Fig 6A). Among these, only 5 genes (GDF15, LCN2, HBEGF, ATF3 and CDKN1A) showed in the qRT-PRC analysis significant p-values (One-way ANOVA test, p<0.05). As to GJA1 and SDC4, qRT-PCR analysis showed no difference between BEAS-C cells and the corresponding derivatives.

In addition, experiments performed with LY294002 indicated that in BEAS-C cells and derivatives the expression of GJA1, SDC4 and CDKN1A was not significantly regulated by modulation of PI3K/AKT activity (Fig 6B). Conversely, the remaining 4 genes GDF15, LCN2, HBEGF and ATF3 showed a significant modulation of their expression upon treatment with LY294002 (Two-way ANOVA test).

On the basis of these results, we propose that GDF15, LCN2, HBEGF, ATF3 and CDKN1A represent important effectors of PI3K/AKT signalling, whose aberrant modulation may contribute to the development of lung cancer, especially when transformation is driven by mutations of PIK3CA or AKT1 or by the loss of PTEN expression. It is important to underline that altought CDKN1A was not apparently modulated by LY294002 it has been recurrently associated to signalling through the PI3K/AKT pathway [40, 41]. For this reason we are regarded CDKN1A as a part of common downstream effectors of PI3K/AKT signalling. CDKN1A encodes the cyclin-dependent kinase inhibitor p21, which promotes cell cycle arrest as an effector of multiple anti-proliferative signals [42, 43]. By phosphorylating p21 at Thr145 and/or Ser146, AKT increases the stability of the protein, induces its cytoplasmic retention [40], thus destroying its cell cycle inhibitory activity and stimulating migration and survival [44–46]. However, in certain conditions p21 may behave as an oncogene [47, 48], with its expression frequently increased in cancer cell lines and tumors [49–52]. In agreement with a pro-oncogenic role of p21, the finding that all the genetic alterations that lead to the activation of the PI3K/AKT pathway induce an increase in the level of p21 mRNA in lung epithelial cells, suggests that p21 may mediate some of the pro-oncogenic activities exerted by aberrant PI3K/AKT signalling in cancer cells such as to stimulate migration and/or invasion or to protect cells from apoptosis [53].

On the other hand, the links between ATF3, GDF15, HBEGF, LCN2 and PI3K/AKT signalling are more uncertain. Previous studies have shown that ATF3, GDF15, HBEGF and LCN2 regulate several cancer-associated functions such as proliferation, migration and invasion, which may also be ascribed to aberrant PI3K/AKT signalling [54–56].

The Activating Transcription Factor 3 (ATF3), a member of the mammalian activation transcription factor/cAMP responsive element-binding (CREB) protein family, is an immediate early gene activated by multiple stress and growth stimuli. ATF3 has been implicated in proliferation and cell survival [57–63], and its expression has been correlated with platinum-sensitivity in NSCLC cells [64].

GDF15 is an ubiquitous cytokine that belongs to the family of transforming growth factor-beta [65], which modulates cell proliferation, differentiation, apoptosis, invasion and metastasis [66] and is over-expressed in many types of cancer [67–69], especially during the transition from localized to metastatic disease [70–75].

Heparin Binding EGF-Like Growth Factor (HBEGF) is an EGF-like ligand of the epidermal growth factor receptor (EGFR) that binds heparin sulfate proteoglycans [76].

LCN2 is an iron-trafficking protein that regulates cell motility and induces epithelial-to-mesenchymal transition [77–79]. Elevated LCN2 expression has been associated with increased tumor cell motility, invasion and survival [80–83].

As anticipated, the links of ATF3, GDF15, HBEGF and LCN2 with the PI3K/AKT pathway are contradictory and, as for ATF3 and GDF15, in most cases have failed to unambiguosly identify these genes as downstream targets of PI3K/AKT signalling [68, 84–86]. The results reported in this manuscript are in line with the known functions of these 4 DEGs. Accordingly, the expression of ATF3, HBEGF, GDF15 and LCN2 is increased in all three derivatives of BEAS-C cells, suggesting that the coordinate changes of these genes may contribute to confer proliferative, pro-migratory, pro-invasive and/or anti-apoptotic properties elicited by aberrant PI3K or AKT in lung epithelial cells. Notably, these results also suggest that similar mechanisms may be operating to confer proliferative, pro-invasive and pro-migratory properties to lung epithelial cells transformed by aberrant PI3K signalling independently of the molecular alteration that have induced it.

Subsequently, we analyzed the BioFunctions enriched by the three lists of exclusive DEGs to investigate whether the different genetic alterations that activate the PI3K/AKT pathway under study here signalled through multiple downstream effectors.

Exclusive DEGs enriched several BioFunctions, including *Cellular Growth and Proliferation*, *Cell Death and Survival*, *Cell Morphology*, *Cell Cycle* and *Protein Synthesis* (see S1–S3 Figs). In BEAS-AKT1-E17K cells, the BioFunctions “Cellular Development” was the most highly enriched and was associated with 8 genes (q-value 7.15*E-03), followed by “Cellular Growth and Proliferation” (13 genes, q-value 7.15*E-03), “Protein Synthesis” (5 genes, q-value 2.66*E-02), “Cell Death and Survival” (6 genes, q-value q-value 2.66*E-02), “Cell Morphology” (5 genes, q-value 2.66*E-02) and “Cell Cycle” (1 gene, q-value 4.13*E-02). We found that active AKT1-E17K regulates expression of genes related to cell cycle progression, cell motility and metastasis such as Transforming growth factor beta receptor 2 (TGFBR2), Cathepsin Z (CTSZ) and Epithelial Membrane Protein 1 (EMP1) [87–89].

In the BEAS-PIK3CA-E545K cells, exclusive DEGs enriched BioFunctions as “Cellular Growth and Proliferation” with 131 genes (q-value 6.02*E-15), “Cellular Movement” (85 genes, q-value 4.35*E-13), “Cellular Development” (115 genes, q-value 5.02*E-11), “Cell Death and Survival” (111 genes, q-value 1.47*E-10), “Cell Morphology” (49 genes, q-value 1.8*E-10), “Cell Cycle” (57 gene, q-value 5.02*E-09) and “DNA Replication, Recombination, and Repair” (21 genes, q-value 4.61*E-05). In particular, we found that mutant PIK3CA modulated the expression of several cycle regulators such as Cyclin D2 (CCND2), Cyclin-dependent kinase 2 (CDK2) and Cdk inhibitors as well as several apoptosis-related genes such as Insulin-like growth factors II (IGFBP2) and Tribbles Pseudokinase 1 (TRIB1) [44, 90–92]. In addition, in agreement with the results of Scrima et al. [8] a high percentage of exclusive DEGs in BEAS-PIK3CA-E545K cells was classified as Transcription Regulators. In fact, in BEAS-PIK3CA-E545K cells, Transcription Regulators were 19 out of 80 exclusive DEGs in the BioFunction *Cell proliferation* of tumor cell lines, 13 in the BioFunction *Invasion of cells* and 9 in the BioFunction *Migration of tumour cell lines* (see S10 File). In BEAS-shPTEN cells, exclusive DEGs showed as enriched Biofunction the “Cell Death and Survival” with 140 genes (q-value 3.16*E-04), “DNA Replication, Recombination, and Repair” (110 genes, q-value 5.31*E-04), “Cellular Growth and Proliferation” (291 genes, q-value 1.71*E-03), “Cell Cycle” (145 genes, q-value 2.93*E-03), “Cell Morphology” (78 genes, q-value 1.85*E-02), “Cellular Development” (220 gene, q-value 2.43*E-02) and “Protein Synthesis” (97 genes, q-value 4.18*E-02).

Finally, the BioFunctions enriched by exclusive DEGs in BEAS-shPTEN cells highlited genes regulating either cell growth such as Asparagine Synthetase (ASNS), wich predicts poor prognosis or invasion such as Four And A Half LIM Domains 2 gene (FHL2) [93, 94]. In the BEAS-shPTEN cells, Transcription Regulators were less frequently represented than in BEAS-PIK3CA-E545K cells, being 26/178 in the BioFunction *Cell proliferation of tumor cell lines* and 8/81 in the BioFunction *Migration of tumour cell lines*. Conversely, in BEAS-shPTEN cells we observed that PTEN regulated two additional categories of genes that were not present in the BEAS-PIK3CA-E545K and BEAS-AKT1-E17K cells, which included Phosphatases in the BioFunction *Cell proliferation of tumor cell lines* (4/178) and *Migration of tumour cell lines* (4/81) and Peptidases in the BioFunction *Cell proliferation of tumor cell lines* (3/178) and *Migration of tumour cell lines* (4/81). See S11 File for detail.

## Conclusions

In summary, in this manuscript we have performed a comparative gene expression analysis of human lung epithelial cells expressing active mutant AKT1 (AKT1-E17K), active mutant PI3KCA (PIK3CA-E545K) or that are silenced for PTEN and have identified and validated a set of genes that may represent downstream targets of constitutive PI3K/AKT signalling in lung cancer cells. Interestingly, loss of PTEN regulates more DEGs than mutant AKT1 (1549 and 133, respectively) whereas the number of DEGs regulated by mutant PIK3CA is intermediate between the former two. Moreover, the transcriptomes of mutant AKT1 and PIK3CA are apparently more similar qualitatively to each other in the Heatmap of Fig 1A in comparison with that induced by PTEN loss. One possibility to justify these results is that PTEN acts upstream PIK3CA that, in turn, acts upstream AKT1.

IPA analysis of the DEGs led to the identification of three relevant BioFunctions enriched by the costitutive activation of AKT1-, PI3K- or PTEN-dependent signalling in lung epithelial cells. The identified BioFunctions include *Proliferation of tumor cell lines* (14 DEGs), *Invasion of cells* (10 DEGs) and *Migration of tumour cell lines* (10 DEGs), with a common core of 5 validated genes (ATF3, CDKN1A, GDF15, HBEGF and LCN2) that may represent novel downstream effectors of the pro-oncogenic activities—proliferation, migration, invasion and/or apoptosis—transmitted through PI3K/AKT signaling.

Notably, the significance of our findings was further strenghtened by two different observations. First, we observed that the pharmacological inhibition of PI3K/AKT signalling in lung epithelial cells modulated the mRNA expression of common DEGs in line with the array results. Second, we observed that in NSCLC cell lines the mRNA expression of these randomly selected down-regulated (SGK1) or up-regulated (GDF15, PTGES, S100P) DEGs correlated with the activation status of the PI3K/AKT pathway. These findings indicate that the identified DEGs may indeed represent critical downstream effectors of the PI3K/AKT signalling axis.

In conclusion, the results reported in this manuscript describe the mRNA profiles elicited in lung epithelial cells by the three most common alterations of the members within the PI3K/AKT pathway and have led to the identification of 5 novel downstream targets of PI3K/AKT signalling that may contribute to confer pro-mitogenic, pro-migratory and pro-invasive properties to cells, once the PI3K/AKT pathway is activated, in a manner that is independent of the molecular alteration that may have caused it. On the other hand, this study led to the identification of genes that are exclusively regulated by mutant AKT1 (TGFBR2, CTSZ, EMP1), mutant PIK3CA (CCND2, CDK2, IGFBP2, TRIB1) and PTEN loss (ASNS, FHL2).

These findings not only shed additional light on the molecular mechanisms that are activated by signalling through the PI3K/AKT pathway in lung epithelial cells, but also may contribute to identify previously unrecognised therapeutic targets in lung cancer driven by aberrant PI3K/AKT signaling.

## Supporting information

S1 Table List of primers for Q-RT-PCR. (DOCX)

S1 Fig Biofunctions enriched by exclusive DEGs in BEAS-AKT1-E17K cells. (TIFF)

S2 Fig Biofunctions enriched by exclusive DEGs in BEAS-PIK3CA-E545K cells. (TIFF)

S3 Fig Biofunctions enriched by exclusive DEGs in BEAS-shPTEN cells. (TIFF)

S1 File List of differentially expressed gene probes in BEAS-AKT1-E17K cells. (XLS)

S2 File List of differentially expressed gene probes in BEAS-PIK3CA-E545K cells. (XLS)

S3 File List of differentially expressed gene probes in BEAS-shPTEN cells. (XLS)

S4 File List of common DEGs between BEAS-PIK3CA-E545K and BEAS-shPTEN cells. (XLSX)

S5 File List of common DEGs between BEAS-PIK3CA-E545K and BEAS-AKT1-E17K cells. (XLSX)

S6 File List of common DEGs between BEAS-shPTEN and BEAS-AKT1-E17K cells. (XLSX)

S7 File List of exclusive DEGs in BEAS-AKT1-E17K cells. (XLSX)

S8 File List of exclusive DEGs in BEAS-PIK3CA-E545K cells. (XLSX)

S9 File List of exclusive DEGs in BEAS-shPTEN cells. (XLSX)

S10 File List of exclusive DEGs that enrich Cell proliferation, Invasion and Migration Biofunctions of tumour cell lines in BEAS-PIK3CA-E545K cells. (XLSX)

S11 File List of exclusive DEGs that enrich Cell proliferation and Migration Biofunctions of tumour cell lines in BEAS-shPTEN cells. (XLSX)
