# Supplementary material for: Specific gene expression signatures induced by the multiple oncogenic alterations that occur within the PTEN/PI3K/AKT pathway in lung cancer
Source: PLoS One. 2017 Jun 29;12(6):e0178865. doi: 10.1371/journal.pone.0178865 (PMC5491004; doi:10.1371/journal.pone.0178865)
Supplement: S1 Table — (DOCX) [file pone.0178865.s001.docx]

| **PRIMER** | **SEQUENCE 5'-3'** |
| --- | --- |
| GDF15 FW | GAGCTGGGAAGATTCGAACA |
| GDF15 REV | AGAGATACGCAGGTGCAGGT |
| PTGES FW | ACGCTGCTGGTCATCAAGA |
| PTGES REV | GGGTCGCTCCTGCAATACT |
| S100P FW | TACCAGGCTTCCTGCAGAGT |
| S100P REV | GTGACAGGCAGACGTGATTG |
| LCN2 FW | CTCCACCTCAGACCTGATCC |
| LCN2 REV | TGCGGGTCTTTGTCTTCTCT |
| SGK1 FW | AGAAATGCTCAGCCTTCCAA |
| SGK1 REV | GAGGAGCCGGTGTACTTCAG |
| IGFBP3 FW | CGCCAGGAAATGCTAGTGAG |
| IGFBP3 REV | GGTGGAACTTGGGATCAGAC |
| PEG10 FW | CCTGTCTTCGCAGAGGAGTC |
| PEG10 REV | CTTCACTTCTGTGGGGATGG |
| MARCKS FW | CTTCAAAGCGAACGGACAG |
| MARCKS REV | GGCTCCTCCTTGTCGGC |
| VWA5A FW | GCCTAACATGAAGCCAGGTC |
| VWA5A REV | ACTCATGGGGCTCTGCATAC |
| BMF FW | GAGGTACAGATTGCCCGAAA |
| BMF REV | TTCAAAGCAAGGTTGTGCAG |
| KRT81 FW | TCAGGGAGTACCAGGAGGTG |
| KRT81 REV | CAGACATTCACAGCCCCAAT |
| DUSP5 FW | TTCCTCAAAGGGGGATATGA |
| DUSP5 REV | GGTTTTCCACACTGGCTGAT |
| ATF3 FW | AAGAGGCGACGAGAAAGAAA |
| ATF3 REV | TCTCCGACTCTTTCTGCAGG |
| HBEGF FW | ACTGAGAGAGACTTGTGCTCA |
| HBEGF REV | GTCTTTCCCCTCTGCAGTCT |
| SDC4 FW | CCCTGAAGTTGTCCATCCCT |
| SDC4 REV | AGTTTCTTGGGTTCGGTGGG |
| GJA1 FW | TAAGCAAAAGAGTGGTGCCC |
| GJA1 REV | CTCCAGCAGTTGAGTAGGCT |
