# Supplementary figures and images for: Specific gene expression signatures induced by the multiple oncogenic alterations that occur within the PTEN/PI3K/AKT pathway in lung cancer
Source: PLoS One. 2017 Jun 29;12(6):e0178865. doi: 10.1371/journal.pone.0178865 (PMC5491004; doi:10.1371/journal.pone.0178865)

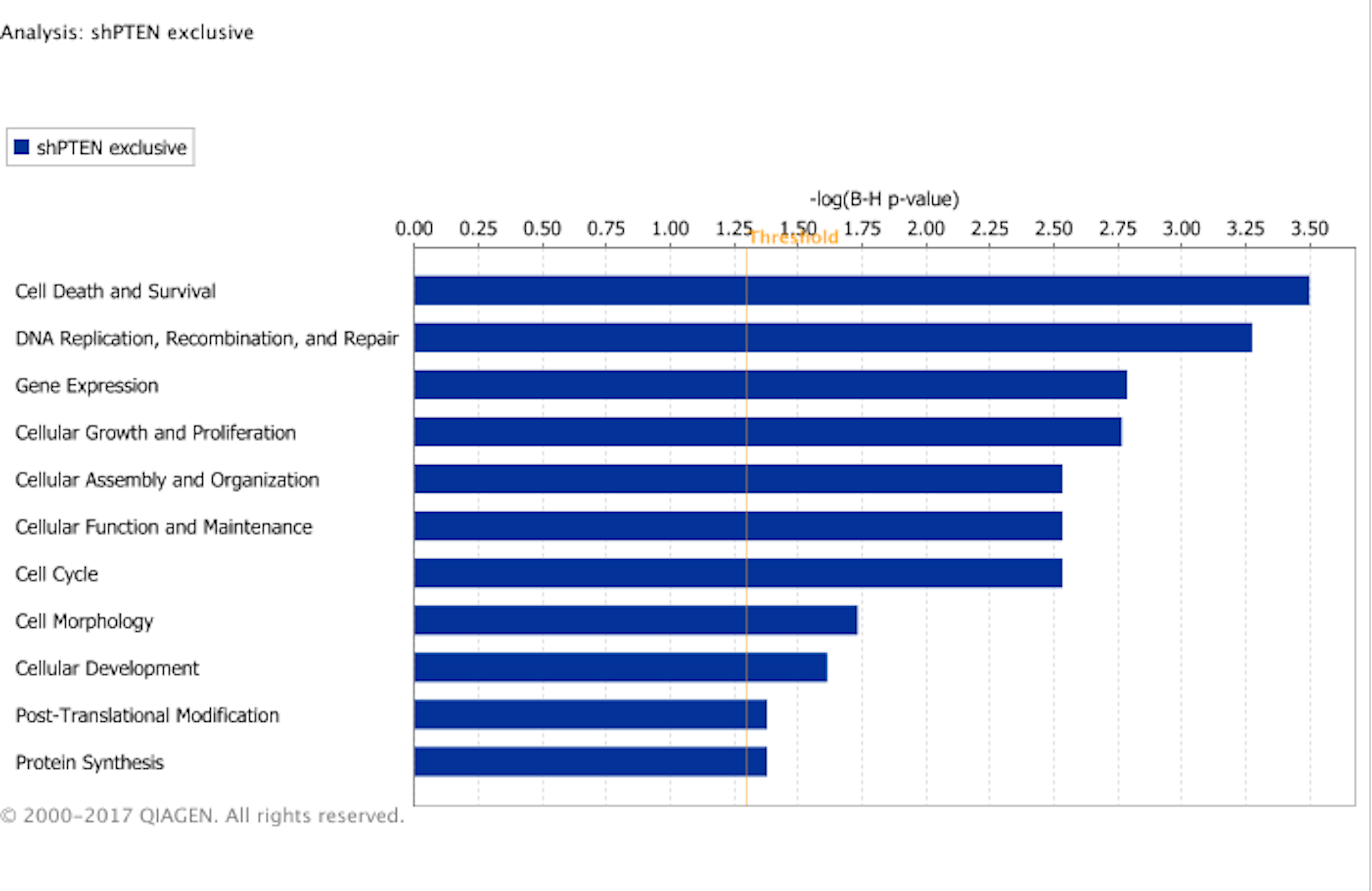

Supplement: S3 Fig — (TIFF) [file pone.0178865.s004.tiff]
